# Mechanisms of gastroprotection of methanol extract of *Melastoma malabathricum* leaves

**DOI:** 10.1186/s12906-015-0638-z

**Published:** 2015-04-28

**Authors:** Zainul Amiruddin Zakaria, Tavamani Balan, Siti Syariah Mamat, Norhafizah Mohtarrudin, Teh Lay Kek, Mohd Zaki Salleh

**Affiliations:** Halal Product Research Institute, Universiti Putra Malaysia, 43400 UPM, Serdang, Selangor Malaysia; Integrative Pharmacogenomics Institute (iPROMISE), Faculty of Pharmacy, Universiti Teknologi MARA, Shah Alam, Selangor Malaysia; Department of Biomedical Science, Faculty of Medicine and Health Sciences, Universiti Putra Malaysia, 43400 UPM Serdang, Serdang, Selangor Malaysia; Department of Pathology, Faculty of Medicine and Health Sciences, Universiti Putra Malaysia, 43400 UPM Serdang, Serdang, Selangor Malaysia

**Keywords:** *Melastoma malabathricum*, Melastomaceae, Methanol extract, Gastroprotective mechanisms, Nitric oxide, Sulfhydryl group, Antioxidant, Anti-inflammatory

## Abstract

**Background:**

*Melastoma malabathricum* L. (Melastomaceae) is a small shrub with various medicinal uses. The present study was carried out to determine the gastroprotective mechanisms of methanol extract of *M. malabathricum* leaves (MEMM) in rats.

**Methods:**

The extract's mechanisms of gastroprotection (50, 250, 500 mg/kg) were studied using the pylorus-ligation in rat model wherein volume, pH, free and total acidity of gastric juice, and gastric wall mucus content were determined. The involvement of endogenous nitric oxide (NO) and sulfhydryl (SH) compounds in the gastroprotective effect of MEMM were also measured. MEMM was subjected to the antioxidant, anti-inflammatory and phytochemical analysis and HPLC profiling.

**Results:**

MEMM contained various phyto-constituents with quercitrin being identified as part of them. MEMM and quercitrin: i) significantly (p < 0.05) reduced the volume and acidity of gastric juice while increasing the pH and gastric wall mucus content.; ii) significantly (p < 0.05) increased the level of SOD, GTP and GTR while significantly (p < 0.05) reduced the level of CAT, MPO and TBARS activities.; iii) exerted gastroprotective activity when assessed using the ethanol-induced gastric ulcer assay, which was reversed by N^G^-nitro-l-arginine methyl esters (L-NAME; an inhibitor of NO synthase) and *N*-ethylmaleimide (NEM; a sulfhydryl (SH) blocker). MEMM inhibited the lipoxygenase (LOX) and xanthine oxidase (XO) activities with the highest affinity for the former while quercitrin showed high affinity for XO activity.

**Conclusions:**

MEMM exhibited a gastroprotective activity due partly to the presence of quercitrin, its antioxidant and anti-inflammatory activities, and via the modulation of NO and SH groups.

## Background

Peptic ulcers are a common disorder of the entire gastrointestinal tract. Of nearly 8 to 10% of the global population affected by peptic ulcers, approximately 5% of them suffer from gastric ulcers [1]. The ulcers that affect the gastrointestinal system are usually aggravated by a disproportion between destructive and defensive factors in the stomach [1]. Although considered as multifactorial disease, it is generally acknowledged that nearly all peptic ulcers are linked to the infection of *Helicobacter pylori* and the major therapeutic aim is to eradicate the *H. pylori* infection. Currently, the first-line medical treatment of gastric ulcer is targeted at eradicating *H. pylori* infection and usually based on triple treatment procedure, which involved the use of gastric ulcer inhibitors (I.e. histamine H_2_-antagonists, proton pump inhibitors, or sucralfate and bismuth) in combination with two types of antibiotics [2]. However, the fact that there are various factors other than *H. pylori* that can trigger gastric ulcer formation should not be ignored [3]. Various approaches have been used in the treatment of gastric ulcer associated with those non-*H. pylori*-related factors such as reducing acid secretion and increasing mucus production but these approaches have been regarded as the second-line treatment.

Despite their effectiveness in eradicating *H. pylori*, the treatment is complex and of high cost, involving the use of at least two antibiotics in combination with gastric acid inhibitors. This combination often causes several unwanted side effects (i.e. antibiotic resistance, recurrence, nausea) [2,3]. Despite the fact that *H. pylori* infections have gradually declined throughout the majority of industrialized countries, a gradual increase in failure of *H. pylori* eradication treatments is observed elsewhere [2]. This is further worsen by the association of those standard antiulcer agents with various unwanted side effects. In consideration of their diverse adverse effects and the occurrence of antibiotic-resistant *H. pylori* strains, the search for new and safe non-antibiotic gastroprotective agents from natural sources, particularly plants, continue to increase all over the world [2,4].

One of the plants that are used to treat gastric ulcer in Malaysia is *Melastoma malabathricum* L. (family Melastomaceae). Locally known to the Malay as ‘*Senduduk*’, *M. malabathricum* is found abundantly in Indian Ocean Island, throughout South and Southeast Asia, Taiwan, China, South Pacific Ocean and Australia. Various parts of the plant are used in Malay traditional medicines to treat a variety of ailments with the leaves, in particular, have been used to treat gastric ulcers among others [5]. Scientifically, the *M. malabathricum* parts have been reported to exhibit various pharmacological activities [5-7]. Other than for its traditional use as antiulcer agent, *M. malabathricum* was chosen in the present study based on the fact that it is one of the famous herbs in Malay medicinal folklore, but received lack of attention among the community. Moreover, *M. malabathricum* has been reported to contain high total phenolic content and to exert high antioxidant and anti-inflammatory activities [5-7], which are important in the mechanisms of antiulcer of any compounds/extracts. It is well known that gastric ulcer, particularly those induce by ethanol, is associated with ROS generation. Ethanol rapidly penetrates the gastric mucosa as it is able to solubilize the protective mucous and causes the release of hydroperoxy-free radicals and superoxide anion. These free radicals cause an increase in oxidative stress in the tissues, which in turn, increases the level of malondialdehyde, a marker of increased lipid peroxidation. Overall, ethanol-induced deleterious effect can be manifested directly via generation of reactive metabolites or indirectly via activation other mechanisms that finally trigger oxidative damage [7]. Hence, extracts/compounds with antioxidants activity play a very important role in scavenging those free radicals and inhibit lipid peroxidation. In lieu of this, *M. malabathricum* has been reported to exert a remarkable antioxidant activity [7] and, therefore, is believed to possess antiulcer potential. Our earlier screening of methanol extract of *M. malabathricum* (MEMM) for antiulcer potential against ethanol- and indomethacin-induced gastric ulcer models has been reported elsewhere [8]. MEMM was found to attenuate ethanol-induced, but aggravated indomethacin-induced, gastric ulcer formation. The ability of MEMM to exert both the gastroprotection and anti-inflammatory activities [5] is in contrast to that of indomethacin, which only exerted anti-inflammatory activity. This observation seems to suggest that the extract activates gastroprotection via a mechanism that is not associated to that of anti-inflammation. Moreover, the anti-inflammatory activity of MEMM might possibly differed from the one exerted by indomethacin. Being an inhibitor of both COXs (i.e. COX-1 and COX-2), indomethacin is more selective for COX-1, which is required for maintaining the protective gastric mucosal layer [9]. The ability of MEMM to intensify indomethacin-induced gastric ulcer formation suggests that MEMM might also inhibited COX-1 action and interfere with the formation of constitutive PG that help to protect the gastric mucosa among others. On the other hand, the ability of MEMM to exert anti-inflammatory activity might be due to its ability to inhibit the COX-2-dependent response associated with the carrageenan-induced rat’s paw edema test [5;9]. Other than that, MEMM might also work contrarily by activating COX-2, leading to increase PGE_2_ synthesis, which has been reported to exert anti-inflammatory activity by binding to one of its receptors, the PGE receptor 4 (EP_4_) [10]. Furthermore, PGE_2_ has been known to be the precursor for the formation of cyclopentenone prostaglandins (cyPG) that exerts anti-inflammatory [11]. Moreover, the ability to activate the PGE_2_ synthesis, which in turn increases the gastric mucus production, can occur via the activation of the EP_3_ receptors [10]. This might again help explain the ability of MEMM to demonstrate anti-inflammatory activity while, at the same time, exert antiulcer activity against the ethanol-, but not indomethacin-induced model. Despite these suggestions, no attempt has been made to determine the possible mechanisms of gastroprotection of MEMM, which could be used to explain the observed antiulcer activity.

Taking into account the above-mentioned report [8] and another reports made by Hussain et al. [6], who assessed the antiulcer potential of aqueous extract of *M. malabathricum* using only one model of gastric ulcer (i.e. ethanol-induced model), the current investigation were designs. Although *M. malabathricum* has never been used in the treatment of *H. pylori* infection, which is supported by its poor antibacterial activity [12,13], the traditional use of the plant for the treatment of gastric ulcer justified the presence research with hope of finding an alternative/natural gastroprotective agent as a replacement to the currently available side effect-baring drugs used in the second-line treatment. Taking this fact into account, the present study aimed to investigate on the possible mechanisms of gastroprotection of MEMM using various rats models.

## Methods

### Chemicals

The chemicals used in this study are of analytical grades and had been prepared immediately before use. The following drugs were used: ranitidine (Sigma Aldrich, USA), quercitrin (Sigma Aldrich, USA), absolute ethanol (Fischer Scientific, USA), N-ethylmaleimide (NEM) (Sigma-Aldrich, USA), N^G^-nitro-l-arginine methyl esters (L-NAME) (Sigma-Aldrich, USA), carbenoxolone (CBX) (Sigma-Aldrich, USA) and diethyl ether (Fischer Scientific, USA).

### Plant materials collection and preparation of MEMM

The leaves of *M. malabathricum* were collected between August and September, 2010 from Serdang, Selangor, Malaysia, and identified by a botanist from the Institute of Bioscience (IBS), Universiti Putra Malaysia (UPM), Serdang, Selangor, Malaysia. A voucher specimen, ACP-0017, has been deposited at the Herbarium of the IBS, UPM, Malaysia. The ground dried leaves (40 g) were soaked three times at room temperature for 24 h with methanol in the ratio of 1:20 (w/v) and the methanol supernatant was evaporated (40°C) under reduced pressure to dryness resulting in a yield of 12.8 g dried and sticky methanol extract (percentage yielded was ≈ 32%).

### Phytochemical screening and HPLC analysis of MEMM

The phytochemical screening and the HPLC analysis of MEMM was performed according to the previously reported method [7]. Phytochemical screening of MEMM was carried out to determine the presence of flavonoids, triterpenes,tannins, alkaloids and saponins according to the conventional protocols as described below.

i) Flavonoids

Approximately 10.0 g of MEMM was separately boiled for 2 to 3 min in 100 ml of water in a water-bath. To 3 ml of the filtrate, 3 ml of acid-alcohol (ethanol:water:concentrated hydrochloric acid in the ratio of 1:1:1), solid magnesium (1 cm) and 1 ml of t-amyl-alcohol were added. The mixtures were then observed for a rose-orange or violate colour change.

ii) Triterpenes

Approximately 1.0 g of MEMM was separately extracted for 24 h in ether. 1 ml of the filtrate was evaporated to dryness and the residue redissolved in several drops of acetic anhydride and then several drops of sulphuric acid were added to the solution. The mixtures were then observed for a green colour change.

iii) Tannins

Approximately 0.2 g of MEMM was separately boiled in 5 ml of water. The mixtures were cooled and filtered. A few drops (3 drops) of 5% ferric chloride solution were added to the filtrate and observed for a blue-black precipitate formation.

iv) Alkaloids

Approximately 0.5 g of MEMM was separately boiled with 10 ml of dilute hydrochloric acid (alcoholic) in a test tube for 5 min. The mixtures were cooled and the debris was allowed to settle. Each of the supernatant liquids was filtered into another test tube and 1 ml of each filtrate was taken into which three drops of Dragendorff’s reagent (potassium bismuth iodide solution) was added, shaken and observed for the appearance of an orange-red spot and a precipitate formation.

v) Saponins

Approximately 0.2 g of MEMM was shaken with water and the mixture was observed for a persistent froth.

The HPLC analysis of MEMM was performed according to the previous report [4] but with slight modifications. In brief, MEMM (10 mg) was suspended in 1 ml methanol. The solution was passed through a filter cartridge (pore size of 0.45 μm) prior to analysis. The filtered sample was analysed using the HPLC system consisting of the Waters Delta 600 with 600 Controller, photodiode array detector (Waters 996). And a Phenomenex Luna (5 μm) (Torrance, CA, USA) column (4.6 mm i.d. × 250 mm). Two solvents denoted as A and B were used for elution of the constituents. A was 0.1% aqueous formic acid and B was acetonitrile. Initial conditions were 95% A and 5% B with a linear gradient reaching 25% B at *t* = 12 min and this condition was maintained for 8 min. B was reduced back to 15% at *t* = 22 min and maintained at this condition for another 8 min (*t* = 30 min). At *t* = 35 min, the programme returned to the initial solvent composition. The flow rate used was 1.0 ml/min and the injection volume was 10 μl. The column oven was set at 27°C and the eluent was monitored at 210, 254, 280, 300, 330 and 366 nm. The retention times, peak areas and UV spectra of the major peaks were analyzed. The HPLC analysis was carried out in the Laboratory of Phytomedicine, Medicinal Plants Division, Forest Research Institute of Malaysia (FRIM), Kepong, Malaysia.

### Experimental animals

Male Sprague Dawley rats (180–200 g; 8–10 weeks old) were obtained from the Veterinary Animal Unit, Faculty of Veterinary Medicine, Universiti Putra Malaysia (UPM), Malaysia and kept under room temperature (27 ± 2°C; 70-80% humidity; 12 h light/darkness cycle) in the Animal Holding Unit, Faculty of Medicine and Health Sciences, UPM. They were supplied with food and water *ad libitum* from the beginning of the experiments. The study protocol of the present study was approved by the Animal House and Use Committee, Faculty of Medicine and Health Sciences, UPM (Ethical approval no: UPM/FPSK/PADS/BR-UUH/00449). The rats were handled in accordance with current UPM guidelines for the care of laboratory animals and the ethical guidelines for investigations of experimental pain in conscious animals [14]. All experiments were conducted between 09.30 and 18.30 h to minimize the effects of environmental changes. Fasting was applied for 48 h prior to all assays wherein the rats were allowed access to only water.

### Determination of the possible mechanisms of gastroprotection of MEMM

#### Pylorus ligation-induced ulceration

Pylorus ligation was carried out according to the method by Shay et al. [15] with slight modifications. Thirty rats were divided randomly into 5 groups (n = 6). Group-I (control) was treated with vehicle (10% DMSO), Group-II (positive control, ranitidine) was given at 100 mg/kg (p.o), Group-III,-IV and-V, rats were treated with MEMM (50, 250 and 500 mg/kg, respectively). Pylorus ligation was performed 1 h after the administration of the test compounds on 48 h fasted rats. Ketamine HCl (100 mg/kg, intramuscular) and xylazine HCl (16 mg/kg, intramuscular) were used to anesthetize the rats prior to ligation of the pylorus. A 2 cm long incision was made in the abdomen just below the sternum on the anesthetized rats. The stomach was exposed, and a thread was passed around the pyloric sphincter and tied in a tight knot. Care was taken while tying the knot to avoid involving blood vessels in the knot. The abdomen was sutured, and the skin was cleaned of any blood spots or bleeding. The animals were sacrificed 6 h after ligation by cervical dislocation.

##### Determination of volume, pH, free and total acidity of gastric content

The stomachs were removed, and the contents were drained out, collected, and centrifuged at 2500 rpm for 10 min. The volume and pH of the gastric juice was measured and was subjected to free and total acidity estimation according to the method described by Srivastava et al. [16]. Free acidity was determined by titration with 0.01 N sodium hydroxide (NaOH) with methyl orange reagent until the color of the solution became yellowish. The volume of alkali added was noted. Then, two to three drops of phenolphthalein was added and the solution was titrated until a definite pink colour appears. The total volume of NaOH added was noted and this corresponds to total acidity. Acidity was calculated using the following formula:

$$ \mathrm{Acidity}=\frac{\mathrm{Volume}\kern0.5em \mathrm{of}\kern0.5em \mathrm{NaOH}\times \mathrm{normality}\kern0.5em \mathrm{of}\kern0.5em \mathrm{NaOH}\times 100}{0.1}\mathrm{m}\mathrm{e}\mathrm{q}/1 $$

##### Estimation of gastric wall mucus content

Gastric wall mucus content was determined by the method described by Corne et al. [17] with slight modifications. The stomach was opened along the greater curvature, weighed, and immersed in 10 ml of 0.1% Alcian Blue in 0.16 M sucrose/0.05 M sodium acetate, pH 5.8 for 2 h. The excessive dye was then removed by two successive rinses in 0.25 M sucrose solution (15 min each). The remaining dye complexed with the gastric mucus were extracted with 0.5 M MgCl_2_ for 2 h and shaken intermittently for 1 min in every 30 min interval. The blue extract was then shaken vigorously with an equal volume of diethyl ether and the resulting emulsion was centrifuged at 3600 rpm for 10 min. The absorbance (OD) of Alcian Blue in the aqueous layer was read at 580 nm using a spectrophotometer. The quantity of Alcian Blue extract per gram wet stomach was then calculated from a standard curve.

##### Ethanol-induced gastric mucosal lesion in L-NAME pre-treated rats

The involvement of endogenous NO in modulating the ethanol-induced gastroprotective activity was determined according to the method of Andreo et al. [18], but with slight modifications. Male rats were randomly divided into twelve groups (n = 6) and fasted for 24 h but allowed free access to water. They were then pre-treated with saline or L-NAME (70 mg/kg; an inhibitor of NO synthase) intraperitoneally (i.p.) and 30 min later, the animals received vehicle (10% DMSO), 100 mg/kg CBX (positive control group) or 500 mg/kg MEMM (p.o.). One hour after the administration of test solutions, gastric ulcer was induced using 5 mL/kg absolute ethanol in all groups. On the other hand, L-Arginine (200 mg/kg) was administered, 30 min after saline or L-NAME treatment and, followed 30 min later by the ethanol administration. All rats were sacrificed 1 hr later by exposure to diethyl ether. The stomach was opened along the greater curvature to determine the ulcer area (UA) as described by Balan et al. [19]. The percentage protection was calculated using the following formula:

$$ \mathrm{Protection}\left(\%\right)=\frac{\left(\mathrm{U}\mathrm{A}\ \mathrm{control}\hbox{--} \mathrm{U}\mathrm{A}\ \mathrm{p}\mathrm{r}\mathrm{e}\hbox{-} \mathrm{treated}\ \mathrm{group}\right)}{\left(\mathrm{U}\mathrm{A}\ \mathrm{control}\right)}\times 100\% $$

##### Ethanol-induced gastric mucosal lesion in NEM pre-treated rats

To investigate the involvement of sulfhydryl (SH) group in the modulation of ethanol-induced gastroprotective activity, the procedures described by Andreo et al. [18] were adopted with slight modifications. The rats were randomly divided into 12 groups (n = 6) and fasted for 24 h but allowed free access to water. The experiment started with pre-treatment (i.p.) of saline or NEM (10 mg/kg), a sulfhydryl (SH-) blocker. Thirty min after the pre-treatment regiment, the rats were administered (p.o.) with vehicle (10% DMSO), 100 mg/kg CBX (positive control group) or 500 mg/kg MEMM followed by the administration of 5 mL/kg ethanol an hour later to induce gastric ulceration. All animals were sacrificed 1 h after receiving ethanol by exposure to diethyl ether. The stomach was removed and gastric damage was determined as described above.

### Biochemical analysis of stomach tissues

Following the macroscopic analyses, superoxide dismutase (SOD) [20], catalase (CAT) [21], myeloperoxidase (MPO) [22], glutathione peroxidase (GTP) [23], glutathione reductase (GTR) [24] and thiobarbituric acid reactive substances (TBARS) [25] enzyme activities in the rat’s stomach tissues were measured. The gastric mucosa was scraped from the antral portion of the stomach using a scrapper and stored at 4°C for biochemical estimation. The scrapped gastric mucosa was subjected to prepare the mucosal homogenate (pH 7.2). The homogenate was then centrifuged at 3000 rpm for 10 min and the supernatant obtained was used for analysis of antioxidant type on ethanol induced gastric mucosal damage. In all antioxidant defense assays, ranitidine (100 mg/kg)-pretreated group was considered as the positive control group.

### Determination of SOD activity

The activity of SOD was determined based on the inhibition of the formation of nicotinamide adenine dinucleotide, phenazine methosulfate and amino blue tetrazolium formazan [20]. Approximately 0.5 ml of tissue homogenate was mixed with 0.4 ml of ethanol and chloroform mixture and centrifuged. To the supernatant, assay mixture (sodium pyrophosphate buffer (0.025 M, pH 8.3), nitroblue tetrazolium, phenazine methosulphate and reduced nicotinamide adenine dinucleotide (NADH)) was added and incubated at 30°C for 90 s. The reaction was arrested by the addition of glacial acetic acid and mixed with *n*-butanol. The intensity of the colour developed in butanol was measured at 560 nm. The SOD activity was measured by the degree of inhibition of this reaction and is expressed as millimole/min/mg protein.

### Determination of CAT activity

The activity of CAT was assayed colorimetrically at 620 nm as described by the method of Sinha [21]. The reaction mixture of 1.5 ml containing 1.0 ml phosphate buffer (0.01 M, pH 7.0), 0.4 ml of 2.0 M H_2_O_2_ and 1.0 ml of tissue homogenate. The reaction was stopped by the addition of 2.0 ml of dichromate–acetic acid reagent (5% potassium dichromate and glacial acetic acid mixture in the ratio of 1:3). Results are expressed as millimole/min/mg protein.

### Determination of MPO activity

The activity of MPO was measured according to the method described by Bradley et al. [22] but with slight modifications. The homogenized samples were frozen and thawed for three times and centrifuged at 1500 g for 10 min at 40°C. Approximately 100 μl of the homogenized supernatant was added to 1.9 ml of 10 mmol/L phosphate buffer (pH 6.0) and 1.0 ml of 1.5 mmol/L O–dianisidine hydrochloride containing 0.0005% (w/v) H_2_O_2_. The absorbance was evaluated at 450 nm on a UV spectrophotometer and the MPO activity in gastric tissues was expressed as μmoles/min/mg protein.

### Determination of GTP activity

The activity of GTP was measured by the method described by Rotruck et al. [23] with slight modifications. The reaction mixture, which contained 0.2 ml of 0.4 M, Tris - HCl buffer, pH 7.0, 0.1 ml of 10 mM sodium azide, 0.2 ml of tissue homogenate (homogenized the tissue in 0.4 M Tris–HCl buffer, pH 7.0), 0.2 ml glutathione and 0.1 ml of 0.2 mM hydrogen peroxide was then incubated at room temperature for 10 min. The reaction was arrested by the addition of 0.4 ml of 10% TCA and subjection to the centrifugation process. The supernatant was assayed for glutathione content by using Ellmans reagent (19.8 mg of 5, 5′–dithiobisnitro benzoic acid (DTNB) in 100 ml of 0.1% sodium nitrate). A molar extinction coefficient of 6.22 × 103 μmol was used to determine the activity of GTP. The enzyme activity was expressed as international units of enzymatic activity/g of protein. International units are expressed as μmoles of hydroperoxides transformed/min/ml of enzyme.

### Determination of GTR activity

The level of GTR was determined by the method of Ellman [24]. Approximately1.0 ml of supernatant was treated with 0.5 ml of Ellmans reagent (19.8 mg of 5, 5′–dithiobisnitro benzoic acid (DTNB) in 100 ml of 0.1% sodium nitrate) and 3.0 ml of phosphate buffer (0.2 M, pH 8.0). The absorbance was read at 412 nm and the GTR activity was expressed as μmoles/min/mg tissue.

### Determination of TBARS content

The extent of LPO was measured by analyzing the levels of TBARS in the gastric mucosa according to the previous method [25] but with minor modification. To 0.5 ml of tissue homogenate, 1.5 ml of 20% acetic acid, 0.2 ml of SDS and 1.5 ml of TBA were added. The mixture was made up to 4 ml with distilled water and heated for 1 hour at 95°C. After cooling, 4.0 ml of butanol–pyridine mixture was added and shaken well. This mixture was then centrifuged at 4000 rpm for 10 min. The organic layer was taken and its absorbance was read at 532 nm and the results were expressed as n mol/g protein.

### Estimation of protein

The protein content in the gastric tissue was estimated according to the method of Lowry et al. [26] but with slight modifications. The tissue sample and the standards (1.0 mg/ml Bovine serum albumin in double distilled water) in different tubes were treated with 5.0 ml of reagent mixture (48% sodium potassium tartarate, 2% copper sulphate and 3% sodium carbonate in 1:48 (v/v)). Then Folin phenol reagent (1:2) was added to the reaction mixture and allowed to stand for 30 min at room temperature. The optical density was read at 710 nm using water as reagent blank.

### In vitro anti-inflammatory activity of MEMM

#### In-vitro effect of MEMM on nitric oxide

##### Cell culture and stimulation

The RAW 264.7 cell line (murine monocytic macrophages) (European Collection of Cell Cultures, Porton Down, UK) was sustained in DMEM supplemented with 10% FBS, 4.5 g/L glucose, L-glutamine (2 mM), sodium pyruvate (1 mM), penicillin (50 U/ml) and streptomycin (50 μg/ml) and 5% CO_2_ at 37°C. The cells (4 × 10^5^ cells/well) were seeded into a 96-well plate and incubated for 2 h at 37°C in a CO_2_ incubator to allow the attachment of cells, and then triggered with stimuli (100 U/ml of IFN-g and 5 μg/ml of LPS) with or without the presence of MEMM in the concentration ranging between 12.5-100 μg/ml. DMSO (vehicle) was used to dissolve the MEMM. The final concentration of DMSO was ensured to be 0.1% in all cultures. Cells were then incubated for 17–20 h at 37°C in a CO_2_ incubator. The NO determination was performed by subjecting the cultured supernatant against the Griess assay and the cells remaining in the well were tested for cell viability assay.

### Nitrite determination

The concentration of nitrite (NO_2_^−^), a stable metabolite of NO in culture medium, was determined using the Griess assay [27]. An equal volume of Griess reagent was mixed with the culture supernatant and the color development was measured at 550 nm. The quantity of nitrite in the culture supernatant was determined based on the standard curve of a sodium nitrite (0–100 μM) freshly prepared in deionized water. Percentage of the NO inhibition was calculated by using the formula below:

$$ \mathrm{NO}\ \mathrm{inhibitory}\left(\%\right)=\frac{{\left[\mathrm{N}{{\mathrm{O}}_2}^{\hbox{-}}\right]}_{\mathrm{control}}*\hbox{--} {\left[\mathrm{N}{{\mathrm{O}}_2}^{\hbox{-}}\right]}_{\mathrm{sample}}}{{\left[\mathrm{N}{{\mathrm{O}}_2}^{\hbox{-}}\right]}_{\mathrm{control}}*}\times 100\% $$

where;

*control is the nitrite level of IFN-γ/LPS-induced group.

### Cell viability

The cytotoxicity of MEMM on the cultured cells was determined by assaying the reduction of 3-(4,5-dimethyl-2-thiazolyl)-2,5-diphenyl-2H-tetrazolium bromide (MTT) [27]. The MTT reagents (0.05 mg/ml) were suspended in sterile PBS, pH 7.0 and then added into each well subsequent to removing the supernatant. This was followed by the incubation of the remaining cells at 37°C for 4 h followed by the addition of 100 μl of 100% DMSO into the wells to dissolve the formazan salts formed. The absorbance was measured at 570 nm. The percentage of cell viability was calculated according to the following formula:

$$ \mathrm{Cell}\ \mathrm{viability}\left(\%\right)=\frac{\mathrm{O}{\mathrm{D}}_{\mathrm{control}}*\hbox{--} \mathrm{O}{\mathrm{D}}_{\mathrm{sample}}}{\mathrm{O}{\mathrm{D}}_{\mathrm{control}}*}\times 100\% $$

where;

*control is the nitrite level of IFN-γ/LPS-induced group

### In vitro effect of MEMM on lipoxygenase activity

The ability of MEMM to exert an inhibitory effect against lipoxygenase activity was determined using the spectrophotometric method [28]. 160 μl sodium phosphate buffer (0.1 M, pH 8.0), 10 μl of MEMM and 20 μl of soybean lipoxygenase solution were combined and incubated for 10 min at 25°C. This was followed by the addition of 10 μl of the sodium linoleic acid solution (substrate) to initiate the reaction. The enzymatic activity converted linoleic acid to (9Z, 11E)-(13S)-13-hydroperoxyoctadeca-9,11-dienoate, which was accompanied by the change of absorbance measured at 234 nm for the period of 6 min. MEMM and reference standards were dissolved in methanol. All reactions were performed in triplicates in a 96-well microplate.

### In vitro effect of MEMM on xanthine oxidase activity

The ability of MEMM to inhibit xanthine oxidase activity was measured using the mspectrophotometric method [29]. 130 μl potassium phosphate buffer (0.05 M, pH 7.5), 10 μl of the MEMM and 10 μl of xanthine oxidase solution were combined and incubated for 10 min at 25°C followed by the initiation of reaction by the addition of 100 μl of xanthine solution (substrate). The enzymatic action converted xanthine to form uric acid and hydrogen peroxides was measured at absorbance of 295 nm. MEMM and reference standards were dissolved in DMSO. All reactions were performed in triplicates in a 96-well UV microplate.

### Evaluation of the pharmacological potential of quercitrin

The ability of quercitrin to exert gastroprotective, antioxidant and anti-inflammatory activities were measured using the respective assay to support its role in the modulation of MEMM gastroprotective activity.

### Statistical analysis

The results obtained were analyzed using One-way analysis of variance (ANOVA), followed by Dunnett’s *post hoc* test or the Newman-Keuls test and expressed as mean ± S.E.M. The statistical software used to analyze the data was SPSS version 15. Results were considered significant when *p <*0*.*05.

## Results

### Phytochemical constituents and HPLC profile of MEMM

The extract demonstrated the presence of flavonoids, triterpenes, tannins, saponins and steroids, but not alkaloids, which is similar to the phytochemicals presence in the dried leaves.

**Figure 1:**
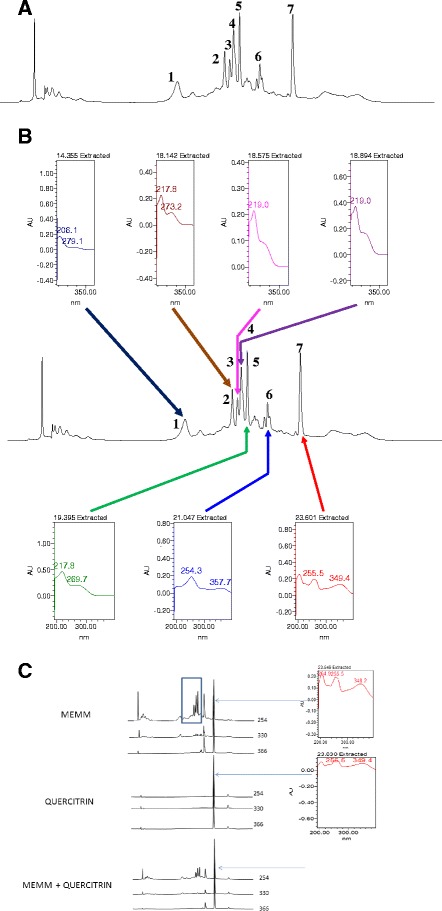
HPLC profile of MEMM at the wavelength 254 nm. **A**. HPLC analysis of MEMM at wavelength 254 nm shows 7 major peaks that were clearly separated at the retention time (R_T_) of 14.355, 18.146, 18.575, 18.894, 19.395, 21.047, and 23.657 min. **B**: Further HPLC analysis was carried out to determine the range of λ_max_ value of the 7 respective peaks detected in MEMM. The chromatogram demonstrates that the λ_max_ value for the six respective peaks falls in the regions of 206.1-279.1, 217.8-273.2, 219.0, 217.8-269.7, 254.3-357.7 and 255.5-349.4 nm, respectively, suggesting, in part the presence of flavonoid-based compounds. Comparison between chromatogram of the standard flavonoid with the chromatogram of MEMM at 300 nm showed the possible presence of quercitrin, which corresponds to peak 7.**C**: Comparison between HPLC chromatogram of MEMM and quercitrin at 300 nm shows that quercitrin was present in MEMM. The peak that was produced by quercitrin corresponds to peak 7 of MEMM.

The phytochemicals analysis of MEMM was carried out using the HPLC method and following several trials at different wavelengths, the phytochemicals profile of MEMM was best isolated at the wavelength of 300 nm (Figure 1A). At the wavelength of 300 nm, 7 major peaks were clearly separated from the MEMM at the retention time (R_T_) of 14.355, 18.146, 18.575, 18.894, 19.395, 21.047, and 23.657 min. In order to predict the class of compounds present in MEMM, further analysis were carried out to determine the range of λ_max_ value of the seven peaks. As can be seen in Figure 1B, the λ_max_ value for the four peaks falls in the regions of 206.1-279.1, 217.8-273.2, 219.8-269.7, 219.0-349.4, 254.3-357.7 and 255.5-349.4 nm, respectively. An attempt to determine the presence of some of the well-known flavonoids shows that only quercitrin was found in the MEMM at the tested wavelength (Figure 1C).

### Possible mechanisms of gastroprotection of MEMM and quercitrin

#### Effects of MEMM on pyloric ligation-induced gastric ulcer: the macroscopic andmicroscopic findings

Table 1 shows the parameters measured from the gastric content following the pyloric ligation-induced gastric ulcer assay. Ranitidine and MEMM exerted significant (p < 0.05) antisecretory activity by reducing the volume of gastric juice collected. In addition, the extract increased the pH of gastric content towards alkaline level and the gastric wall mucus content while reducing the total and free acidity when compared to the control group. Quercitrin, at 50 and 250 mg/kg, also exerted gastroprotective activity by significantly (p < 0.05) increasing the pH and, total and free acidity. Moreover, quercitrin also significantly (p < 0.05) increased the gastric wall mucus production (Table 1).

**Table 1:** **Effects of MEMM on the parameters of gastric juice and on gastric wall mucus content obtained from pylorus-ligature rats**

**Model**	**Treatment**	**Dose, (mg/kg)**	**Gastric juice**				**Gastric wall mucus**
			**Volume (ml)**	**pH**	**Total acidity (meq/l)**	**Free acidity (meq/l)**	**Alcian blue** **μg/g wet tissue**
Pylorus ligation	10% DMSO	-	5.83 ± 1.01	1.50 ± 0.09	5427.00 ± 380.40	4367.00 ± 573.30	85.68 ± 13.76
	Ranitidine	100	1.63 ± 0.30^a^	4.41 ± 0.76^b^	1433.00 ± 92.15^c^	1500.00 ± 235.20^d^	241.30 ± 14.11*
	MEMM	50	6.50 ± 0.52	1.82 ± 0.12	3100.00 ± 392.30^c^	2420.00 ± 438.80^d^	151.26 ± 32. 17*
		250	2.58 ± 0.61^a^	2.43 ± 0.22^b^	3107.00 ± 458.00^c^	2040.00 ± 327.60^d^	354.24 ± 38.71*
		500	1.95 ± 0.16^a^	3.55 ± 0.63^b^	3105.00 ± 435.2^c^	933.30 ± 291.90^d^	422.08 ± 67.73*
	Quercitrin	50	4.31 ± 0.74^a^	2.38 ± 0.23^b^	4648.00 ± 223.50^c^	3211.00 ± 261.60^d^	258.63 ± 17.48*
		250	4.72 ± 0.38^a^	4.43 ± 0.16^b^	3519.00 ± 380.40^c^	2367.00 ± 382.80^d^	407.42 ± 21.76*

^a,b,c,d,^Data with different superscript differed significantly (p < 0.05) when compared to the 10% DMSO-pretreated group within the respective column.

*Data differed significantly (p < 0.05) when compared to the 10% DMSO-pretreated group within the respective column.

### Ethanol-induced gastric mucosal lesion in L-NAME or NEM pre-treated rats

**Figure 2:**
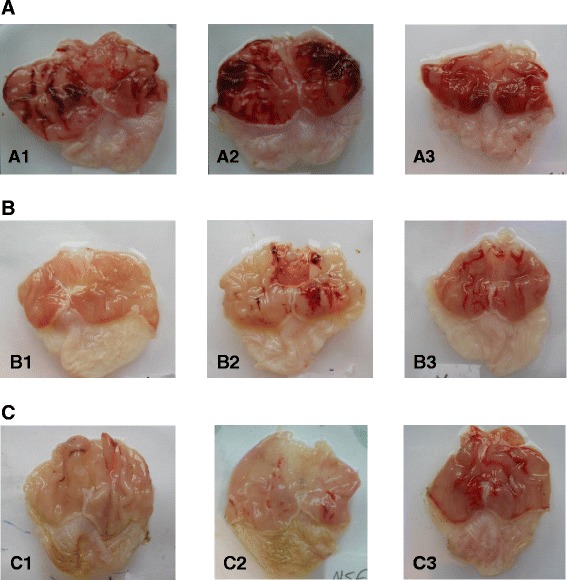
Gross examination of the gastric mucosa of rats treated with 10% DMSO-, CBX- or MEMM following pretreatment with saline, L-NAME or NEM, respectively. **A**. Gross examination of the gastric mucosa in rats. A1, A2 and A3 represent the control (10% DMSO) group pre-treated with saline, L-NAME (70 mg/kg) or NEM (10 mg/kg), respectively. Pre-treatment with L-NAME or NEM in control group aggravated the lesion formation as compared to saline pre-treated control group. **B**: Gross examination of the gastric mucosa in rats treated with CBX (100 mg/kg). B1, B2 and B3 represent the positive control group pre-treated with saline, L-NAME (70 mg/kg) or NEM (10 mg/kg), respectively. Pre-treatment with L-NAME or NEM in positive control group significantly reversed the gastroprotection activity of CBX.**C**: Gross examination of the gastric mucosa in rats treated with MEMM (500 mg/kg). C1, C2 and C3 represent the groups pre-treated with saline, L-NAME (70 mg/kg) or NEM (10 mg/kg), respectively. The gastroprotective effect exerted by MEMM against ethanol-induced damage was reversed by the pre-treatment of L-NAME or NEM, suggesting the involvement of nitric oxide and sulfhydryl compounds in the gastroprotection conferred by MEMM.

As shown in Table 2 and Figure 2, pre-treatment with L-NAME and NEM augmented the ulcerative index induced by ethanol in comparison to group pre-treated with saline in 10% DMSO-treated group (Figure 2A(1–3)). Moreover, pre-treatment with L-NAME and NEM caused significant (p < 0.05) reduction in CBX- induced gastroprotection (Figure 2B(1–3)). On the other hand, pre-treatment with L-NAME and NEM caused significant (p < 0.05) increase in the ulcer area formation when compared to their respective counterpart pre-treated with normal saline (Figure 2C(1–3)). Quercitrin exerted significant (p < 0.05) gastroprotective activity, which was also significantly (p < 0.05) attenuated by L-NAME and NEM (2D(1–3)).

**Table 2:** **Effects of the MEMM on gastric lesions induced by ethanol in rats pretreated with L- NAME or NEM**

**Pre-treatment**	**Treatment**	**Dose, mg/kg**	**Ulcer area (mm** ^**2**^ **)**
Saline	10% DMSO	-	33.33 ± 1.84
	CBX	100	3.17 ± 0.31^a^
	MEMM	500	0.83 ± 0.40^a^
	Quercitrin	250 mg/kg	1.62 ± 0.74^a^
L-NAME	10% DMSO	-	49.38 ± 3.64^b^
	CBX	100 mg/kg	7.00 ± 0.76^ce^
	MEMM	500 mg/kg	5.25 ± 0.98^cf^
	Quercitrin	250 mg/kg	22.48 ± 1.93^cg^
NEM	10% DMSO	-	61.50 ± 4.62^b^
	CBX	100 mg/kg	12.00 ± 2.46^dh^
	MEMM	500 mg/kg	54.33 ± 4.82^di^
	Quercitrin	250 mg/kg	38.85 ± 5.21^dj^

Values are mean ± SEM (n = 6 rats/group).

One-way ANOVA followed by Student–Newman–Keuls.

^a,b^Data differed significantly (p < 0.05) when compared to the (saline + 10% DMSO)- pretreated group.

^c^Data differed significantly (p < 0.05) when compared to the (L-NAME + 10% DMSO)- pretreated group.

^d^Data differed significantly (p < 0.05) when compared to the (NEM + 10% DMSO)- pretreated group.

^e,h^Data differed significantly (p < 0.05) when compared to the (saline + CBX)-pretreated group.

^f,i^Data differed significantly (p < 0.05) when compared to the (saline + MEMM)-pretreated group.

^g,j^Data differed significantly (p < 0.05) when compared to the (saline + quercitrin)-pretreated group.

### Effect of MEMM on biochemical parameters of stomach tissue

Further attempt was also made to investigate the role of enzymatic and non-enzymatic antioxidant defenses on the ulceration development in all gastric tissues wherein the antioxidant levels of SOD, CAT, MPO GTP and GTR activity were determined. Table 3 shows the effect of MEMM on the level of SOD, CAT and MPO enzymes in rats subjected to the ethanol-induced gastric ulcer. The gastric ulcer induced group (negative control) showed significant (P < 0.05) decrease in SOD activity in comparison to the normal control (ethanol-untreated) group, which was reversed by the 250 and 500 mg/kg MEMM, and 30 mg/kg lansoprazole. As for the CAT level, the negative control group significantly (P < 0.05) increased the enzyme level but was reduced by both concentrations of MEMM as well as lansoprazole. On the other hand, the MPO level significantly (P < 0.05) increased in ethanol-administrated group in comparison to the normal control group. Pretreatment with MEMM, at 250 and 500 mg/kg, or 30 mg/kg lansoprazole significantly (P < 0.05) reduced the MPO level, which increases by ethanol administration. Quercitrin significantly (p < 0.05) increased the level of antioxidant enzymes namely SOD but reduced the level of CAT and MPO (Table 3).

**Table 3:** **Effect of methanol extract of** ***M. malabathricum*** **leaves (MEMM), quercitrin and ranitidine on levels of superoxide dismutase (SOD), catalase (CAT) and myeloperoxidase (MPO) enzymes in rats of ethanol-induced gastric tissues**

**Experimental groups**	**SOD (mmol/min/mg of protein** ^**−1**^ **)**	**CAT (mmol/min/mg protein** ^**−1**^ **)**	**MPO (UI mg of protein** ^**−1**^ **)**
Normal (Untreated)	53.26 ± 2.93	5.04 ± 0.87	1.76 ± 0.28
Negative Control (Ethanol)	38.73 ± 4.81*	7.17 ± 0.72*	3.52 ± 0.23*
100 mg/kg Ranitidine + Ethanol	58.72 ± 5.80^#^	5.11 ± 0.74^#^	2.23 ± 0.18^#^
250 mg/kg MEMM + Ethanol	42.38 ± 3.25	6.08 ± 0.59	2.88 ± 0.45^#^
500 mg/kg MEMM + Ethanol	50.31 ± 6.06^#^	5.32 ± 0.31^#^	2.11 ± 0.28^#^
50 mg/kg Qurcitrin + Ethanol	55.83 ± 4.22^#^	5.18 ± 1.01^#^	1.94 ± 0.63^#^
250 mg/kg Qurcitrin + Ethanol	52.89 ± 5.09^#^	5.41 ± 0.83^#^	1.61 ± 0.19^#^

*significant at p < 0.05 when compared against the normal (untreated) group using the Student’s t-test.

^#^significant at p < 0.05 when compared against the negative control (ethanol-treated) group using the ANOVA followed by Dunnett’s test.

Table 4 shows the effect of MEMM on the level of GSH, namely GTP and GTR, and TBARS in rats subjected to the ethanol-induced gastric ulcer. Rats in the negative control group exhibited significant (P < 0.05) decrease in GTP and GTR levels when compared to the normal untreated group. On the other hand, MEMM, at the dose of 250 and 500 mg/kg, was found to reverse the ethanol effect and caused significant (P < 0.05) increase in GTP level but decrease in GTR level. The same observations were not seen with the standard drug, 30 mg/kg lansoprazole. Ethanol alone was found to significantly (P < 0.05) increase the TBARS level when compared to the normal untreated control group wherein both doses of MEMM, but not 30 mg/kg ranitidine, significantly (P < 0.05) reversed the ethanol-induced increases in TBARS level. Quercitrin also significantly i(p < 0.05) increased the level of antioxidant exzymes GTP and GTR while at the same time reduced the level of non-enzymatic oxidative component such as m TBARS (Tables 3 and 4).

**Table 4:** **Effect of methanol extract of** ***M. malabathricum*** **leaves (MEMM), quercitrin and ranitidine on levels of glutathione peroxidase (GTP), glutathione reductsase (GTR) and thiobarbituric acid reactive substances (TBARS) in rats of ethanol-induced gastric tissues**

**Experimental groups**	**GTP (nmol min** ^**−1**^ **mg of protein** ^**−1**^ **)**	**GTR (nmol min** ^**−1**^ **mg of protein** ^**−1**^ **)**	**TBARS (nmol TBARS** ^**−1**^ **mg of protein** ^**1**^ **)**
Normal (Untreated)	316.22 ± 52.77	28.37 ± 0.91	2.81 ± 0.23
Negative Control (Ethanol)	127.83 ± 20.84*	12.74 ± 0.77*	6.27 ± 0.56*
100 mg/kg Ranitidine + Ethanol	186.81 ± 26.73^#^	14.39 ± 1.96	4.13 ± 1.07^#^
250 mg/kg MEMM + Ethanol	271.56 ± 41.31^#^	15.62 ± 1.01^#^	4.29 ± 0.33^#^
500 mg/kg MEMM + Ethanol	310.18 ± 30.07^#^	18.86 ± 2.34^#^	3.01 ± 0.92^#^
50 mg/kg Qurcitrin + Ethanol	201.16 ± 13.45^#^	17.33 ± 0.84^#^	3.17 ± 0.12^#^
250 mg/kg Qurcitrin + Ethanol	308.24 ± 22.87^#^	25.76 ± 1.41^#^	2.25 ± 0.33^#^

The results were expressed as mean ± SEM.

*significant at p < 0.05 when compared against the normal (untreated) group using the Student’s t-test.

^#^significant at p < 0.05 when compared against the negative control (ethanol-treated) group using the ANOVA followed by Dunnett’s test.

### *In vitro* anti-inflammatory activity of MEMM

#### Cell viability and NO production

**Figure 3:**
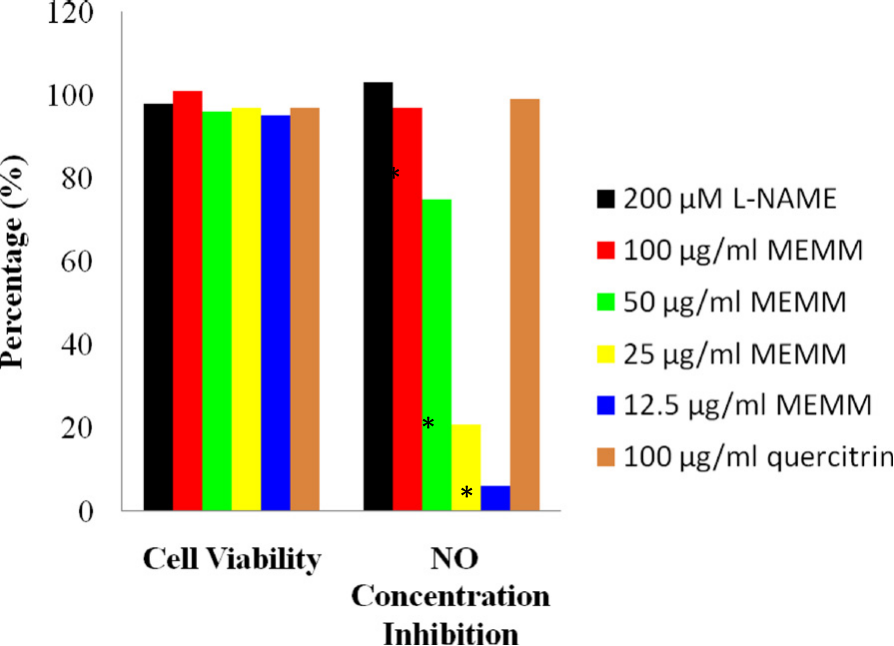
Effect of MEMM on the nitric oxide (NO) production and RAW 264.7 cells’ viability. The assays were done in triplicate. One way ANOVA was followed by Dunnett’s post hoc test, *p < 0.05 shows significant difference as compared to the IFN-γ/LPS-treated group (inflammation induced group). 200 μM L-NAME.

The in vitro effect of MEMM (12.5-100 μg/ml) on percentage of RAW 264.7 cell viability and NO production determined using the MTT assay is shown in Figure 3. The results showed that the MEMM, at all concentrations, did not affect cell viability. The extract was found to significantly (p < 0.05) increased the percentage of inhibition of NO production in LPS-induced RAW 264.7 cells. The amount of nitrite, a stable metabolite of NO, was used as the indicator of NO production in the medium. L-NAME, a standard NOS inhibitor, caused a significant inhibition of NO (*p* < 0.05). Quercitrin, on the other hand, also did not affect cell viability but caused significant (p < 0.05) inhibition of NO production.

### Effect of MEMM on lipoxygenase and xanthine oxidase activities

MEMM was found to exert anti-inflammatory activity when assessed using the in vitro lipoxygenase and xanthine oxidase assays. At the concentration of 100 μg/ml, MEMM showed high inhibitory activity against the lipoxygenase assay, but low inhibitory effect against the xanthine oxidase assay (Table 5). Quercitrin exerted high inhibitory effect against xanthine oxidase activity, but not lipo-oxygenase activity (Table 5).

**Table 5:** **Effect of MEMM and quercitrin on the** ***in vitro*** **lipoxygenase and xantine oxidase activities**

**Sample**	**Concentration (** **μg/ml)**	**Lipoxygenase (%)**	**Xanthine oxidase (%)**
MEMM	100	82.12 ± 3.29 (H)	32.53 ± 4.50 (L)
Quercitrin	100	9.07 ± 1.12 (L)	58.79 ± 6.11 (M)

All values are expressed as mean ± SEM.

Note: H-high (71-100%); M-moderate (41-70%); L-low (0-40%); NA-not active.

## Discussion

The present study aimed to investigate the gastroprotective mechanisms of MEMM using the pylorus ligation model. This is one of the most widely used gastroprotective model to study the effect of drugs on gastric acid and mucus secretion. An increase in gastric hydrochloric acid (HCl) secretion and/or stasis of acid cause auto digestion of the gastric mucosa and breakdown of the gastric mucosal barrier in the pylorus-ligated animals [30]. Agents that reduce secretion of gastric aggressive factors such as pepsin and acid (antisecretory) and/or increase mucin secretion (cytoprotective) are effective gastroprotective agents [10]. In the present study, the ability of MEMM to exert gastroprotective effect was proven via the extract ability to reduce the pH and acidity (total and free), but increase the volume of gastric juice secretion.

Moreover, the extract was also able to increase the release of gastric wall mucus secretion. Mucus is secreted by the mucus neck cells and coats the gastric mucosa [31]. There are several mechanisms involved to increase gastroprotection via increased mucus secretion, which include improving the buffering of acid in gastric juice, lessening of stomach wall friction during peristalsis and gastric contractions, and by acting as an effective barrier to back diffusion of hydrogen ions [32]. The ability of MEMM to increase the amount of gastric mucus wall content proves that enhancement of the gastric mucosal defense action could be one of the possible mechanism of gastroprotection exerted by MEMM.

In addition, the role of nitric oxide (NO) and sulfhydryl group in the modulation of gastroprotective activity exerted by MEMM was also proven using the ethanol-induced gastric ulcer model. The gastroprotective effect exerted by MEMM against ethanol-induced damage was reversed by the pre-treatment of L-NAME, an inhibitor of NO synthase, suggesting that the low level of NO triggered by L-NAME administration reduced the extract’s gastroprotective potential. This finding indicates the important and possible participation of NO in the gastroprotective effect shown by MEMM. NO is considered one of the most important defensive endogenous agents in the gastric mucosa [33]. NO plays an important role in the modulation of gastric mucosal integrity against hyperacidity or exposure to ulcerogens and in regulating acid secretion [34]. It also helps in maintaining the gastric mucosal blood flow, barrier function and alkaline production [35].

On the other hand, pre-treatment of NEM, a SH blocker, alone increased the area of ulcer formation when assessed using the ethanol-induced gastric ulcer model and, when pre-challenged with MEMM reversed the extract’s gastroprotective potential. These findings suggested that the sulfhydryl group is important in the attenuation of gastric ulcer formation and that the absence of sulfhydryl group following the NEM administration reduce the extract’s ability to exert gastroprotective activity. These observations suggest that the gastroprotection showed by MEMM depends on the presence of mucosal SH compound levels. Endogenous SH compounds are key agents in mucosal protection against ethanol-induced gastric injury [36]. The development of ethanol-induced gastric damage is accompanied by a decrease in mucosal sulfhydryl compounds because these compounds are neutralized when they bind to the free radicals produced by injured tissues [37]. Therefore, the SH compounds provide protective effects through binding free radicals formed following the ethanol treatment and by controlling the production of mucus [38]. In addition, SH compounds are also involved in the maintenance of the mucus disulfide bridges that connects the mucus subunits. In the condition where the disulfide bridges are damaged, the mucus would become more soluble, resulting in a gastric mucosa that is more susceptible to injuries [39].

Moreover, various mechanisms of defense against toxicity and tissue damage triggered by ROS have been reported. These mechanisms involving enzymatic and non-enzymatic systems have been investigated and their involvements are proven. The enzymatic and non-enzymatic antioxidant defenses comprise SOD, CAT, GTP, GTR, β-tocopherol, vitamin C, β-carotene and vitamin A. These antioxidants also take part in the prevention of gastric damage [23,40,41]. SOD, regarded as the first line of defense against the harmful effects of oxygen radicals in the cells, forages ROS by catalyzing the dismutation of superoxide to H_2_O_2_ [42]. Previous study reported that ethanol depresses SOD activities [43] leading to failure of the body defense mechanism to convert superoxide radicals to H_2_O_2_. It is suggested that inhibition of SOD activity cause an increase flux of superoxide radicals in cellular compartments, which may explain the increased in lipid peroxidative indices in the present study. CAT, a highly reactive enzyme that reacts with H_2_O_2_ to form water and molecular oxygen, plays a protective antioxidant effect against the deleterious actions of lipid peroxidation. Ethanol increased CAT activity when given alone, which was reversed following pretreatment with MEMM or ranitidine. The increased CAT activity in group treated only with ethanol could be related to increase formation of H_2_O_2_ from superoxide radicals by SOD. Thiobarbituric acid reactive substances (TBARS) are byproduct of lipid peroxidation, which is produce upon oxidative injury induced by ethanol. Excessive generation of free radicals (i.e. hydroxyl ethyl radical, superoxide radical (O^2−^), hydroxyl radical (OH^−^), peroxyl radical and hydrogen peroxide) occur as a result of ethanol administration [44]. These free radicals have a high tendency to react swiftly with lipids, thus, triggering lipid peroxidation, a process that can lead to membrane disorganization and consequently reduces in membrane fluidity [45]. The ethanol-induced increases in TBARS level was reversed by MEMM and ranitidine suggesting reduction in the lipid peroxidation activity upon the oral administration of MEMM or the standard drug. Glutathione (GT), a powerful nucleophilic antioxidant, plays multiple roles such as serving as an electron donor for certain antioxidative enzymes, maintaining cells in a reduced state, and critical for cellular protection (i.e. detoxification of ROS, control of inflammatory cytokines and, conjugation and excretion of toxic molecules) [46]. Reduction of GTR in tissues initiates impairment of the cellular protections against ROS and may result in peroxidative injury [47]. Administration of ethanol alone was found to triggered reduction in the level of GTR while pretreatment with MEMM or ranitidine reversed the ethanol effect and, therefore, increase the GTR level. This finding indicates the ability of MEMM to restore the reduced GTR levels for efficient cellular defense against the action of free radicals. GTP, on the other hand, is essentials in the elimination of H_2_O_2_ and lipid hydroperoxides in gastric mucosal cells and also plays role in the maintenance of a constant ratio of reduced glutathione to oxidized glutathione in the cell [48]. GTP activity was reduced in group that received only ethanol but was reversed by MEMM or ranitidine suggesting that the gastroprotective activity of MEMM may appear through glutathione metabolism. Lastly, myeloperoxidase (MPO) is a sensitive and specific tissue marker enzyme for acute inflammation due to neutrophil infiltration in various gastric injuries [49]. Tissue MPO increased in ethanol-administered rat stomach tissues in comparison to the normal untreated rats. This increase in MPO activity may be linked to increase in the levels of neutrophil infiltration and H_2_O_2_ in the ethanol-induced gastric damaged tissues. In the present study, MEMM and ranitidine demonstrated a tendency to counter the increase in MPO level caused by ethanol. Other than the modulation of enzymatic and non-enzymatic antioxidant defenses as described above, the extract potential as antioxidant agent may also contributes to the observed gastroprotective activity [7]. Various reports have showed the effectiveness of pretreatment of scavengers of reactive oxygen species in attenuating most of the gastric mucosal injury suggesting that the antioxidant effect is one of the most important mechanisms of gastroprotective activity [50,51].

The ability of MEMM to exert anti-inflammatory activity was proven via its ability to exert inhibitory action against the LOX and XO activities. The important of LOX in the attenuation of gastric ulcer has been reported elsewhere [52], as the leucotrienes play an important role in blood coagulation and GIT irritation [53]. Thus, inhibition of 5-LOX will be helpful in attenuating the formation of gastric ulcer during long term therapy of non-selective and COX-1 selective NSAID’s.

The gastroprotective potential of MEMM could also be attributed to the presence of various phytochemical constituents isolated and identified from different parts of *M. malabathricum*. In the present study, we have shown the presence of at least quercitrin in MEMM using the HPLC method. We have tested and showed the ability of quercitrin to exert antiulcer, antioxidant and anti-inflammatory activities. These findings were constant to previous reports on pharmacological activities of quercitrin. Quercitrin has been reported to exert intestinal anti-inflammatory effect via inhibition of iNOS expression [54]. Later, quercitrin, following conversion into quercetin after glycoside’s cleavage by the intestinal microbiota, was reported to exert in vivo anti-inflammatory activity in the experimental model of rat colitis induced by dextran sulfate sodium via the inhibition of the NF-jB pathway [55]. Recently, quercitrin, isolated from *Ixora coccinea*, was reported to exert remarkable antioxidant activity with low IC_50_ when assessed using the DPPH radical scavenging and nitric oxide scavenging assays [56]. Moreover, quercitrin, isolated from *Panicum virgatum*, has been reported to inhibit lipid peroxidation when assessed using the thiobarbituric acid reactive substances (TBARS) assay [57]. Based on those reports, it is plausible to suggest that the antiulcer activity of MEMM was partly attributed to the anti-inflammatory and antioxidant activities of quercitrin present in the extract.

## Conclusion

In conclusion, our present study shows that the MEMM exerted gastroprotective activity through various mechanisms, including the antisecretory and cytoprotective activities and, the enzymatic (such as SOD, CAT, GTP and myeloperoxidase) and non-enzymatic (such as anti-lipid peroxidation) antioxidant defense mechanisms. Moreover, modulation of NO and SH compounds also contributes to the gastroprotection mechanism conferred by MEMM. In addition, the high antioxidant activity, high total phenolic content and synergistic activity of various phytochemcial constituents could also be another mechanism in which MEMM exerts its protective activity. Further studies are being carried out in our laboratory to isolate and identify the bioactive compound(s) responsible for the gastroprotective activity of MEMM.

## References

[1] Calam J, Baron JH (2001). Pathophysiology of duodenal and gastric ulcer and gastric cancer. Brit Med J.

[2] Gasparetto M, Pescarin M, Guariso G. Helicobacter pylori Eradication Therapy: Current Availabilities. Int Schol Res Network Gastroenterol 2012, 2012: Article ID 186734, 8 pages doi:10.5402/2012/186734 10.5402/2012/186734 PMC3414051 22900197

[3] Yakoob J, Jafri W, Jafri N, Islam M, Abid S, Hamid S (2005). Prevalence of non-Helicobacter pylori duodenal ulcer in Karachi, Pakistan. World J Gastroenterol.

[4] Zakaria ZA, Abdul Hisam EE, Mohtarruddin N, Rofiee MS, Othman F, Hasiah AH (2012). Methanol extract of *Bauhinia purpurea* leaf possesses antiulcer activity. Med Princ Pract.

[5] Mohd. Joffry S, Yob NJ, Rofiee MS, MeorMohd. Affandi MMR, Othman F, Abdah MA et al. Melastoma malabathricum (L.) Smith: a review of its ethnomedicinal, chemical and pharmacological uses. Evid-Bsed Compl Alt 2012, Article ID 258434. doi:10.1155/2012/258434. 10.1155/2012/258434 PMC3254175 22242040

[6] Hussain F, Abdulla MA, Mohd Noor S, Ismail S, Mohd Ali H (2008). Gastroprotective effects of *Melastoma malabathricum* aqueous leaf extract against ethanol-induced gastric ulcer in rats. Am J Biochem Biotechnol.

[7] Zakaria ZA, Rofiee MS, Mohamed AM, Teh LK, Salleh MZ (2011). In vitro antiproliferative and antioxidant activities, and total phenolic contents of the extracts of *Melastoma malabathricum* leaves. J Acupunct Meridian Stud.

[8] Zabidi Z, Wan Zainulddin WN, Mamat SS, Shamsahal Din S, Kamisan FH, Yahya F (2012). Antiulcer activity of methanol extract of *Melastoma malabathricum* leaves in rats. Med Princ Pract.

[9] Guay J, Bateman K, Gordon R, Mancini J, Riendeau D (2004). Carrageenan-induced paw edema in rat elicits a predominant prostaglandin E2 (PGE2) response in the central nervous system associated with the induction of microsomal PGE2 synthase-1. J Biol Chem.

[10] Tang EHC, Libby P, Vanhoutte PM, Xu A (2012). Anti-inflammation therapy by activation of prostaglandin EP4 receptor in cardiovascular and other inflammatory diseases. J Cardiovasc Pharmacol.

[11] Rossi A, Kapahi P, Natoli G, Takahashi T, Chen Y, Karin M (2000). Anti-inflammatory cyclopentenone prostaglandins are direct inhibitors of IkappaB kinase. Nature.

[12] Wiart C, Mogana S, Khalifah S, Mahan M, Ismail S, Buckle M (2004). Antimicrobial screening of plants used for traditional medicine in the state of Perak, Peninsular Malaysia. Fitoterapia.

[13] Sunilson JAJ, James J, Thomas J, Jayaraj P, Varatharajan R, Muthappan M (2008). Antibacterial and wound healing activities of *Melastoma malabathricum* Linn. Afr J Infect Dis.

[14] Zimmermann M (1983). Ethical guidelines for investigations of experimental pain in conscious animals. Pain.

[15] Shay H, Komarov SA, Fels SS, Meranze D, Gruenstein M, Siplet H (1945). A simple method for the uniform production of gastric ulceration in the rat. Gastroenterol.

[16] Srivastava V, Viswanathaswamy AH, Mohan G (2010). Determination of the antiulcer properties of sodium cromoglycate in pylorus-ligated albino rats. Indian J Pharmacol.

[17] Corne SJ, Morrisey SM, Woods RJ (1974). A method for the quantitative estimation of gastric barrier mucus. J Physiol.

[18] Andreo MA, Ballesteros KVR, Hiruma-Lima CA, Machado da Rocha LR, Souza Brito ARM, Vilegas W (2006). Effect of *Mouriri pusa* extracts on experimentally induced gastric lesions in rodents: Role of endogenous sulfhydryls compounds and nitric oxide in gastroprotection. J Ethnopharmacol.

[19] Balan T, Sani MHM, Suppaiah V, Mohtarrudin N, Suhaili Z, Ahmad Z, Zakaria ZA (2013). Antiulcer activity of methanol extract of *Muntingia calabura* leaves involves the modulation of endogenous nitric oxide and non-protein sulfhydryl compounds. Pharm Biol.

[20] Kakkar P, Das B, Viswanathan PN (1984). A modified spectrophotometric assay of superoxide dismutase. Indian J Biochem Biophys.

[21] Sinha KA (1972). Colorimetry assay of Catalase. Anal Biochem.

[22] Bradley PP, Priebat DA, Christensen RD, Rothstein G (1982). Measurement of cutaneous inflammation: estimation of neutrophil content with an enzyme marker. J Invest Dermatol.

[23] Rotruck JT, Pope AL, Ganther HE, Swanson AB (1973). Selenium: biochemical roles as a component of glutathione peroxidase. Science.

[24] Ellman GL (1959). Tissue sulfhydryl groups. Arch Biochem Biophys.

[25] Okhawa H, Oishi N, Yagi K (1979). Assay for lipid peroxides in animal tissue by thiobarbituric acid reaction. Anal Biochem.

[26] Lowry OH, Rosebrough NL, Farr AL, Randall RJ (1951). Protein measurement with Folin Phenol reagent. J Biol Chem.

[27] Lee KH, Padzil AM, Syahida A, Abdullah N, Zuhainis SW, Maziah M (2011). Evaluation of anti-inflammatory, antioxidant and antinociceptive activities of six Malaysian medicinal plants. J Med Plant Res.

[28] Azhar-Ul-Haq, Malik A, Anis I, Khan SB, Ahmed E, Ahmed Z, Nawaz SA, Choudhary MI (2004). Enzymes Inhibiting Lignans from *Vitex negundo*. Chem Pharmaceut Bull.

[29] Noro T, Miyase T, Kuroyanagi M (1983). Monoamine oxidase inhibitor from the rhizomes of *Kaempferia galanga* L. Chem Pharmaceut Bull.

[30] Kumar A, Singh V, Chaudhary AK (2011). Gastric antisecretory and antiulcer activities of *Cedrusdeodara* (Roxb.) Loud. In Wistar rats. J Ethnopharmacol.

[31] Goel RK, Bhattacharya SK (1991). Gastroduodenal mucosal defence and mucosal protective agents. Ind J Exp Biol.

[32] Venables CW (1986). Mucus, pepsin and peptic ulcer. Gut.

[33] Brzozowski T, Konturek PC, Sliwowski Z, Kwiecień S, Drozdowicz D, Pawlik M (2006). Interaction of nonsteroidal anti-inflammatory drugs (NSAID) with *Helicobacter pylori* in the stomach of humans and experimental animals. J Physiol Pharmacol.

[34] Tariq M, Khan HA, Elfaki I, Arshaduddin M, Al Moutaery M, Al Rayes H (2007). Gastric antisecretory and antiulcer effects of simvastatin in rats. J Gastroenterol Hepatol.

[35] Calatayud S, Barrachina D, Esplugues JV (2001). Nitric oxide: relation to integrity, injury, and healing of the gastric mucosa. Microsc Res Tech.

[36] Szabo S, Vattay P (1990). Experimental gastric and duodenal ulcers. Advances in pathogenesis. Gastroenterol Clin North Am.

[37] Maity S, Vedasiromoni JR, Ganguly DK (1998). Role of glutathione in the antiulcer effect of hot water extract of black tea (*Camellia sinensis*). Jpn J Pharmacol.

[38] Salim AS (1993). Sulfhydryl-containing agents: new approach to the problem of refractory peptic ulceration. Pharmacology.

[39] Avila JR, de la Lastra CA, Martin MJ, Motilva V, Luque I, Delgado D (2005). Role of endogenous sulphydryls and neutrophil infiltration in the pathogenesis of gastric mucosal injury induced by piroxicam in rats. Inflamm Res.

[40] Goel RK, Sairam K, Rao V (2001). Role of gastric antioxidant and antihelicobacter pyroli activities in antiulcerogenic activities of Plantain banana (Musa spaientum var. paradisiaca). Indian J Exp Biol.

[41] Mates JM, Perez-Gomez C, Nudez de Castro I (1999). Antioxidant enzymes and human diseases. Clin Biochem.

[42] Okado-Matsumoto A, Fridovich I (2001). Subcellular distribution of superoxide dismutases (SOD) in rat liver: Cu-, Zn-SOD in mitochondria. J Biol Chem.

[43] Rukkumani R, Aruna K, Varma PS, Rajasekaran KN, Menon VP (2004). Comparative effects of curcumin and an analog of curcumin and PUFA induced oxidative stress. J Pharm Pharm Sci.

[44] Schlorff EC, Husain K, Somani SM (1999). Dose- and time-dependent effects of ethanol on plasma antioxidant system in rat. Alcohol.

[45] Recknagel RO, Ghoshal AK (1996). Quantitative estimation of peroxidative degeneration of rat liver microsomal and mitochondrial lipids after carbon tetrachloride poisoning. Exp Mol Pathol.

[46] Brown LA, Harris FL, Ping XD, Gauthier TW (2004). Chronic ethanol ingestion and the risk of acute lung injury: a role for glutathione availability?. Alcohol.

[47] Jaya DS, Augstine J, Menon VP (1993). Role of lipid peroxides, glutathione and antiperoxidative enzymes in alcohol and drug toxicity. Indian J Exp Biol.

[48] Halliwell B, Gutteridge JMC, Cross CE (1992). Free radicals, antioxidants, and human disease: where are we now?. J Lab Clin Med.

[49] Bayir Y, Odabasoglu F, Cakir A, Aslan A, Suleyman H, Halici M (2006). The inhibition of gastric mucosal lesion, oxidative stress and meutrophil infiltration in rats by the lichen constituent diffractaic acid. Phytomedicine.

[50] Mota KSL, Dias GEN, Pinto MEF, Luiz-Ferreira Â, Monteiro Souza-Brito AR, Hiruma Lima CA (2009). Flavonoids with gastroprotective activity. Molecule.

[51] Trivedi NP, Rawal UM (2001). Hepatoprotective and antioxidant property of *Andrographis paniculata* (Nees) in BHC induced liver damage in mice. Indian J Exp Biol.

[52] Fiorucci S, Distrutti E, de Lima OM, Romano M, Mencarelli A, Barbanti M (2003). Relative contribution of acetylated cyclo-oxygenase (COX)-2 and 5-lipooxygenase (LOX) in regulating gastric mucosal integrity and adaptation to aspirin. FASEB J.

[53] Pelletier JM, Lajeunesse D, Reboul P, Pelletier JP (2003). Therapeutic role of dual inhibitors of 5-LOX and COX, selective and nonselective non-steroidal anti-inflammatory drugs. Ann Rheum Dis.

[54] Camuesco D, Comalada M, Rodriguez-Cabezas ME, Nieto A, Lorente MD, Concha A (2004). The intestinal anti-inflammatory effect of quercitrin is associated with an inhibition in iNOS expression. Br J Pharmacol.

[55] Comalada M, Camuesco D, Sierra S, Ballester I, Xaus J, Glvez J (2005). *In vivo* quercitrin anti-inflammatory effect involves release of quercetin, which inhibits inflammation through down-regulation of the NF-jB pathway. Eur J Immunol.

[56] Bose S, Maji S, Chakraborty P (2013). Research article quercitrin from *Ixora coccinea* leaves and its anti-oxidant activity. J PharmaSciTech.

[57] Uppugundla N, Engelberth A, Vandhana Ravindranath S, Clausen EC, Lay JO, Gidden J, Carrier DJ (2009). Switchgrass water extracts: extraction, separation and biological activity of rutin and quercitrin. J Agric Food Chem.

